# Effectiveness of inactivated influenza vaccine against laboratory-confirmed influenza among Chinese elderly: a test-negative design

**DOI:** 10.1186/s12877-024-05003-3

**Published:** 2024-05-07

**Authors:** Tianchi Yang, Ling Tang, Pingping Li, Baojun Li, Lixia Ye, Jifang Zhou

**Affiliations:** 1 Institute of Immunization and Prevention, Ningbo Municipal Center for Disease Control and Prevention, Ningbo, Zhejiang China; 2 Ningbo Health Information Center, Ningbo, Zhejiang China; 3 Jiangbei District Center for Disease Control and Prevention, Ningbo, Zhejiang China; 4 Haishu District Center for Disease Control and Prevention, Ningbo, Zhejiang China; 5 School of International Pharmaceutical Business, China Pharmaceutical University, Jiangsu, China

**Keywords:** Influenza, Vaccine effectiveness, Healthcare visit, Test-negative, Elderly

## Abstract

**Background:**

Evidence on the effectiveness of influenza vaccination in the elderly is limited, and results are controversial. There are also few reports from China.

**Methods:**

We conducted a test-negative case-control study design to estimate influenza vaccine effectiveness (VE) against laboratory-confirmed influenza-associated visits among elderly (aged ≥ 60 years) across four influenza seasons in Ningbo, China, from 2018 to 19 to 2021-22. Influenza-positive cases and negative controls were randomly matched in a 1:1 ratio according to age, sex, hospital, and date of influenza testing. We used logistic regression models to compare vaccination odds ratios (ORs) in cases to controls. We calculated the VE as [100% × (1-adjusted OR)] and calculated the 95% confidence interval (CI) around the estimate.

**Results:**

A total of 30,630 elderly patients tested for influenza with virus nucleic acid or antigen during the study period. After exclusions, we included 1 825 influenza-positive cases and 1 825 influenza-negative controls. Overall, the adjusted VE for influenza-related visits was 63.5% (95% CI, 56.3–69.5%), but varied by season. Influenza VE was 59.8% (95% CI, 51.5–66.7%) for influenza A and 89.6% (95% CI, 77.1–95.3%) for influenza B. The VE for ages 60–69 and 70–79 was 65.2% (95% CI, 55.4–72.9%) and 69.8% (95% CI, 58.7–77.9%), respectively, but only 45.4% (95% CI, 6.2–68.2%) for ages 80 and over.

**Conclusions:**

Standard-dose inactivated influenza vaccine has shown good protection in the elderly in China. However, protection may not be satisfactory in people aged 80 years and older.

**Supplementary Information:**

The online version contains supplementary material available at 10.1186/s12877-024-05003-3.

## A summary of the article’s main point

The inactivated influenza vaccine has demonstrated favorable protection among the Chinese elderly population, irrespective of strain matching. Nonetheless, caution is warranted as the standard-dose inactivated vaccine may offer unsatisfactory protection in individuals aged 80 years and above.

**Supplementary Information:**

The online version contains supplementary material available at 10.1186/s12877-024-05003-3.

## Introduction

Influenza is a severe public health problem of global concern, leading to roughly 3–5 million severe cases and 290,000-650,000 respiratory deaths globally each year, most of which are in the elderly, especially in patients with chronic underlying illnesses, who are at increased risk of hospitalization and death due to complications from influenza [1–3]. Vaccination is the most effective measure to prevent influenza or reduce its morbidity and mortality [1, 4–6]. However, controversy persists regarding the effectiveness of influenza vaccines and the value of their application in different target groups [7, 8]. In addition, there is still limited evidence of the effectiveness of influenza vaccination in preventing influenza morbidity in the elderly, and there are heterogeneous results on the protective effect [9, 10]. Even fewer studies have been reported from China [11]. Just one study in Beijing reported a modest protective effect of the 2013–2014 influenza vaccine among the elderly, which was not statistically significant [12]. Consistent long-term studies are required to evaluate the protective effect of the influenza vaccine among the Chinese elderly.

Although Chinese health authorities recommend annual influenza vaccination for the elderly, in China, except for a few regions, influenza vaccination is self-funded, and the vaccination coverage is well below the World Health Organization’s recommended target of 75% coverage. With government funding, Ningbo City implemented a voluntary and free vaccination program for trivalent inactivated influenza vaccine (IIV) during the period of September to November 2020. This initiative targeted all elderly individuals aged 70 and above with Ningbo residency. Subsequently, in the subsequent period of September to November 2021, the eligibility criteria for this free vaccination policy were expanded to include individuals aged 65 and older with Ningbo residency. Following the implementation of this free vaccination policy, the influenza vaccination coverage among the elderly population aged 60 and above in the local area experienced a remarkable surge. Specifically, the vaccination rate increased significantly, from below 5% during the 2019–2020 influenza season to approximately 40% during the 2021–2022 season.

In this context, using a test-negative design, we conducted a retrospective observational study to estimate the vaccine effectiveness (VE) of IIV among senior residents aged 60 years and older from the 2018-19 to 2021-22 influenza season based on records of influenza A/B virus antigen testing by gold immunochromatography assay or RNA testing by polymerase chain reaction (PCR) assay in the Ningbo regional clinic information system.

## Methods

We conducted a retrospective test-negative case-control study of elderly aged 60 and older in Ningbo, China, from 2018 to 19 to 2021-22 influenza seasons. Ningbo is adjacent to Shanghai, with a resident population of more than 9 million in 2020. As a coastal city in southeastern China, seasonal influenza in Ningbo is prevalent in winter and spring and tends to peak in summer [13]. During the 2018-19 to 2021-22 influenza seasons, influenza vaccination began each year in September. Therefore, each influenza season in the study is defined as October 1 to September 30 of the following year, divided into two epidemic stages: October to April and May to September. Ethics approval was obtained from the Ethics Committee of Ningbo Municipal Center for Disease Control and Prevention, China (IRB. No: 202,208). The need for Informed Consent was waived by the Ethics Committee of Ningbo Municipal Center for Disease Control and Prevention, China.

### Subjects enrollment and laboratory diagnosis

Eligible subjects were elderly residents aged 60 years and older with influenza-like illness (ILI, see Supplemental Table 1) who visited hospitals or community health service centers in Ningbo and had a test for detecting influenza A/B virus antigen by gold immune chromatography assay or detecting influenza A/B virus RNA by PCR assay between October 1, 2018 and September 30, 2022. Patients were excluded if they had any of the following: (1) no identification number, (2) less than one year of continuous enrollment in the regional clinic information system before testing, (3) no vaccination record retrieved, (4) developing an illness within 14 days of vaccination, (5) received two or more doses of influenza vaccine during the same influenza season, (6) took anti-influenza viral medication within seven days before testing, (7) repeated detection of the same pathogen within 30 days.

In China, laboratory confirmed influenza is a nationally notifiable condition, and general practitioners routinely collect specimens for influenza testing. All 65 public hospitals and 154 primary care organizations from 10 districts in Ningbo provided influenza A/B virus antigen testing service by gold immune chromatography or RNA testing by PCR assay. In this study, a test-negative design was used to estimate the effectiveness of the influenza vaccine. Laboratory-confirmed influenza cases were elderly medically-attended ILIs who tested positive for influenza by gold immune chromatography or PCR assay. Controls were elderly medically-attended ILIs who tested negative for influenza. To ensure that our findings were not biased by a priori systematic demographic differences between cases and controls, cases and controls were frequency matched 1:1 regarding sex, age, medical institutions providing influenza testing, and influenza test date.

### Data collection

This study is based on the data warehouse of the Ningbo Regional Health Information Platform (NRHIP), which collects and integrates electronic health records from hospitals and community clinics in the region. In 2016, the platform reached the top standardization and maturity measurement of the National Health Commission’s regional health information connectivity. By 2019, the platform had covered all 65 public hospitals and 154 primary care organizations. Vaccination data of residents in Ningbo from all regional clinics are recorded electronically and transmitted to the platform in real time. As a rule, access to healthcare and vaccinations in the study area requires registering a citizen’s unique personal identification number. The NRHIP uses the identification number to centralize all information in an individual’s health record. In this study, the NRHIP provided each participant basic demographic information, disease diagnoses, influenza test information, and vaccination records. Multiple studies have demonstrated the reliability and accuracy of the data sources [14–16].

Since the attenuated influenza vaccine is unavailable for adults in China, we restricted the VE estimates to inactivated vaccines, including trivalent and quadrivalent ones. A participant was defined as vaccinated if he or she had received a dose of influenza vaccine at least 14 days before the onset of influenza-related symptoms in the respective influenza year [7].

### Statistical analyses

We used medians and interquartile ranges (IQRs) or frequencies and percentages to represent continuous or categorical variables, respectively. Demographic characteristics, underlying disease characteristics, and other potential confounders were compared between cases and controls and between vaccinated and unvaccinated participants using the Pearson χ^2^ test or Fisher’s exact test for categorical variables and the Wilcoxon rank sum test or t-test for continuous variables. Unconditional multiple logistic regression model was adopted to assess the odds ratio (OR) of vaccination among influenza-positive and negative patients after adjusting household registration (a unique Chinese system, tracks and manages individuals’ residency status, serving as a vital document for citizen identification, demographic recording, and the facilitation of social welfare services.), the status of chronic underlying disease, influenza vaccination in the previous year, and influenza season. These factors were used to adjust as potential confounders as they were associated with influenza positivity and vaccination status.

VE was calculated as (1- OR) × 100%. VE analysis was performed for influenza overall and by virus type (subtypes A and B), age group, influenza season, and epidemic stage. All estimates were not further calculated for adjusted VE if the number of vaccinated cases after stratification was less than 5. All statistical tests were two-sided and considered statistically significant at *P* < 0.05 or the lower 95% confidence interval limit for VE > 0. All analyses were conducted using R3.4.2 software.

### Subgroup and sensitivity analyses

We conducted subgroup analyses by adding a stratifying variable as an independent covariate and in an interaction term with case status. We identified statistically significant differences in the VE estimates across strata using the 2-tailed *P* value at a significance level of < 0.05 for the interaction term. We applied the final multiple logistic regression model to a redefined study cohort for sensitivity analyses.

## Results

**Fig. 1:**
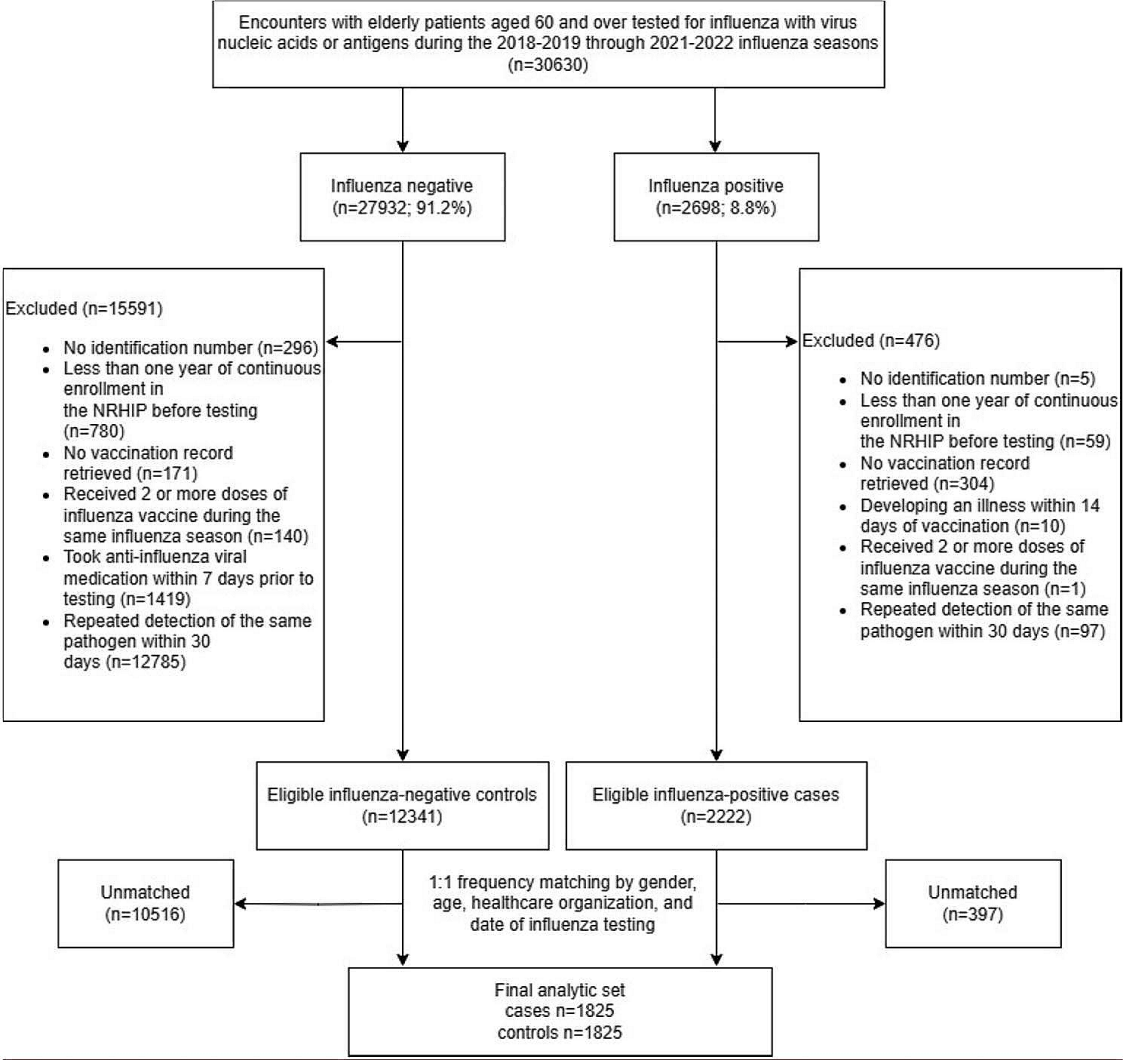
Consort Diagram for Study Enrollment

We identified 30,630 elderly patients aged 60 and over tested for influenza during the 2018-19 through 2021-22 influenza seasons. After exclusions and sampling, we included 1825 influenza-positive cases (1587 tested positive for influenza A, 200 tested positive for influenza B, and 38 tested untyped) and 1825 influenza-negative controls in the entire cohort (Fig. 1).

The median age was 67.0 years (IQR, 9); 1758 (48.2%) were male, and 3362 (92.1%) were local domiciles. The most common underlying comorbidities was hypertension (1230 cases, 33.7%), followed by diabetes (432 cases, 11.8%). There were no differences between influenza-positive and influenza-negative older adults regarding age, sex, or comorbidities such as diabetes mellitus, cardiovascular disease, or neoplasms. Compared to those who tested negative for influenza, those who tested positive for flu were less likely to have hypertension (31.9% vs. 35.5%, *P* = 0.025), local domicile (91.2% vs. 93.0%, *P* = 0.037), and to have been vaccinated in the season before inclusion (12.2% vs. 17.3%, *P* < 0.001) (Table 1).

**Table 1:** Demographic and Clinical Characteristics of Study Participants by Case Status

Characteristic		Total (*N* = 3650)	Cases (*N* = 1825)	Controls (*N* = 1825)	*P* value ^a^
Age at presentation, y	M (IQR)	67 (9)	66 (9)	67 (10)	0.612
Age group, y					1.000
	60 to < 70	2392 (65.5)	1196 (65.5%)	1196 (65.5%)	
	70 to < 80	934 (25.6)	467 (25.6%)	467 (25.6%)	
	80+	324 (8.9)	162 (8.9%)	162 (8.9%)	
Sex					1.000
	Female	1892 (51.8)	946 (51.8%)	946 (51.8%)	
	Male	1758 (48.2)	879 (48.2%)	879 (48.2%)	
Permanent residents	Yes	3362 (92.1)	1664 (91.2%)	1698 (93.0%)	0.037
Influenza season					1.000
	2018-19	436 (12.0)	218 (11.9%)	218 (11.9%)	
	2019-20	578 (15.8)	289 (15.8%)	289 (15.8%)	
	2020-21	180 (4.9)	90 (4.9%)	90 (4.9%)	
	2021-22	2456 (67.3)	1228 (67.3%)	1228 (67.3%)	
Vaccination during the enrollment season	Yes	931 (25.5)	320 (17.5%)	611 (33.5%)	< 0.001
Vaccination in the previous season	Yes	538 (14.7%)	222 (12.2%)	316 (17.3%)	< 0.001
Medical condition					
Diabetes mellitus	Yes	432 (11.8)	219 (12.0%)	213 (11.7%)	0.759
Cerebrovascular diseases	Yes	71 (2.0)	35 (1.9%)	36 (2.0%)	0.905
Hypertension	Yes	1230 (33.7)	583 (31.9%)	647 (35.5%)	0.025
Tumors	Yes	92 (2.5)	47 (2.6%)	45 (2.5%)	0.833

Abbreviations: M (IQR), median (interquartile range).

^a^ Cases and controls were compared using the Pearson χ2 test or Fisher’s exact test for categorical variables and the Wilcoxon rank sum test or t-test for continuous variables.

The majority of seniors included in the final analysis were not vaccinated against influenza during the enrollment season (2719, 74.5%), while 860 seniors (23.6%) received the 3-valent inactivated vaccine and 71 (1.9%) received the 4-valent inactivated vaccine. The vaccinated elderly did not differ from the unvaccinated elderly during the enrollment season regarding gender, cardiovascular diseases, or tumors. Vaccination rates varied by season (*P* < 0.001). Compared with unvaccinated elderly (*P* < 0.001), vaccinated elderly were older, more likely to be of local domicile (98.7% vs. 89.8%, *P* < 0.001), more likely to have diabetes mellitus (15.6% vs. 10.6%, *P* < 0.001) or hypertension (45.8% vs. 29.6%, *P* < 0.001), and more likely to have been vaccinated in the season before inclusion (37.1% vs. 7.1%, *P* < 0.001) (see Supplemental Table 2).

### Vaccine effectiveness

Of the 1825 cases and 1825 controls used to assess VE, 320 (17.5%) influenza-positive cases and 611 (33.5%) influenza-negative controls received inactivated seasonal influenza vaccine. The adjusted VE for inactivated vaccine against influenza visits during 2018–2022 was 63.5% (95% CI, 56.3–69.5%). The adjusted VEs for influenza A and B were 59.8% (95% CI, 51.5–66.7%) and 89.6% (95% CI, 77.1–95.3%), respectively (Table 2).

**Table 2:** Inactivated Influenza Vaccine Effectiveness Against Medical Consultations Among People Aged 60 Years and Older, 2018-19 to 2021-22

VE by Characteristic	Cases who were vaccinated, No./Total No. (%)	Controls who were vaccinated, No./Total No. (%)	Estimated VE^a^ (95% CI), %	
			Unadjusted	Adjusted ^b^
Overall	320/1825 (17.5)	611/1825 (33.5)	57.8 (50.7 to 63.8)	63.5 (56.3 to 69.5)
Permanent residents	315/1664 (18.9)	604/1698 (35.6)	57.7 (50.5 to 63.9)	64.3 (57.2 to 70.3)
Virus Type				
Influenza A-infected cases	311/1587 (19.6)	547/1587 (34.5)	53.7 (45.5 to 60.6)	59.8 (51.5 to 66.7)
Influenza B-infected cases	9/200 (4.5)	56/200 (28.0)	87.9 (74.7 to 94.2)	89.6 (77.1 to 95.3)
By age group, y				
60 to < 70	107/1196 (9.0)	263/1196 (22.0)	65.1 (55.6 to 72.6)	65.2 (55.4 to 72.9)
70 to < 80	172/467 (36.8)	286/467 (61.2)	63.1 (51.9 to 71.7)	69.8 (58.7 to 77.9)
80+	41/162 (25.3)	62/162 (38.3)	45.3 (12.1 to 66.0)	45.4 (6.2 to 68.2)
By season				
2018-19	2/218 (0.9)	15/218 (6.9)	87.5 (44.5 to 97.2)	83.0 (21.6 to 96.3)
2019-20^c^	8/289 (2.8)	34/289 (11.8)	78.6 (53.0 to 90.3)	74.1 (41.3 to 88.6)
2020-21	6/90 (6.7)	28/90 (31.1)	84.2 (59.5 to 93.8)	92.8 (75.5 to 97.9)
2021-22^c^	304/1228 (24.8)	534/1228 (43.5)	57.2 (49.2 to 64.0)	61.5 (53.2 to 68.3)
Time of the season				
October to April	14/549 (2.6)	76/549 (13.8)	83.7 (70.8 to 90.9)	83.3 (68.4 to 91.2)
May to September	306/1276 (24.0)	535/1276 (41.9)	56.3 (48.2 to 63.1)	62.0 (53.8 to 68.7)
Medical condition				
Diabetes mellitus				
No	267/1606 (16.6)	519/1612 (32.2)	57.9 (48.3 to 65.6)	62.4 (52.6 to 70.1)
Yes	53/219 (24.2)	92/213 (43.2)	56.9 (45.0 to 66.2)	64.4 (52.5 to 73.2)
Cerebrovascular diseases				
No	312/1790 (17.4)	599/1789 (33.5)	58.1 (50.9 to 64.1)	63.7 (56.5 to 69.7)
Yes	8/35 (22.9)	12/36 (33.3)	40.7 (-69.4 to 79.3)	- ^d^
Hypertension				
No	175/1242 (14.1)	330/1178 (28.0)	58.0 (50.3 to 64.5)	62.6 (54.6 to 69.1)
Yes	145/583 (24.87)	281/647 (43.4)	58.0 (36.6 to 72.2)	69.5 (49.9 to 81.4)
Tumors				
No	312/1778 (17.6)	596/1780 (33.5)	57.7 (50.5 to 63.9)	63.7 (56.4 to 69.7)
Yes	8/47 (17.0)	15/45 (33.3)	59.0 (-9.4 to 84.6)	- ^d^

Abbreviations: VE, vaccine effectiveness; CI, confidence interval

^a^ Calculated as (1 - odds ratio [OR]) × 100%, where OR is the odds of vaccination among cases compared with the odds of vaccination among controls

^b^ Adjusted for factors identified in Table 1 and Supplemental Table 2 as having significant bivariate associations with vaccination or case status, such as age at the time of testing, household registration, season, vaccination status in the season before inclusion, and prevalence of diabetes mellitus or hypertension

^c^ The influenza vaccine is poorly matched to the prevalent influenza A(H3N2) and B virus strains during the 2019-20 season. The influenza vaccine is poorly matched to the prevalent influenza B(Victoria) virus strain during the 2021-22 season

^d^ The adjusted estimate is not available due to the small sample size of the subgroup

### Subgroup analyses

Influenza VE varied by season, with the highest in the 2020-21 season (92.8%; 95% CI, 75.5–97.9%) and the lowest in the 2021-22 season (61.5%; 95% CI, 53.2–68.3%; Table 2). The VE point estimates for the 60–69 and 70–79 age groups were similar at 65.2% (95% CI, 55.4–72.9%) and 69.8% (95% CI, 58.7–77.9%), respectively. The VE point estimate for the 80 and above age group was lower (45.4%; 95% CI, 6.2–68.2%), but the confidence interval for the VE estimate was larger due to the smaller number of people in this age group. VE varied significantly during different periods of the influenza season, including 83.3% (95% CI, 68.4–91.2%) during the October-April period and 62.0% (95% CI, 53.8–68.7%) during the May-September period. The overall VE estimate was 84.2% before the coronavirus disease 2019 (COVID-19) epidemic compared with 61.9% during the COVID-19 epidemic. Restricting the analysis to the pre-COVID-19 pandemic reduces the sample size so substantially that the results of the stratified studies are not statistically significant at *P* < 0.05. However, the VE point estimates for influenza A were close before and after the COVID-19 epidemic (Supplemental Table 3). Due to insufficient numbers, VE estimates for the quadrivalent IIV could not be calculated

### Sensitivity analyses

Restricting the analysis to those tested for influenza virus nucleic acid or excluding patients with untyped influenza from the case group did not significantly change the estimates of VE. In addition, when we restricted the analysis to outpatients or the local household population, the adjusted overall VE for influenza vaccine was 63.3% and 64.3%, respectively, consistent with the primary analysis results. Finally, we restricted the study period to the peak of the influenza epidemic, and the results showed no significant changes in VE estimates for overall, influenza A and influenza B (Table 3)

**Table 3:** Sensitivity Analyses of Vaccine Effectiveness of Inactivated Influenza Vaccine Among People Aged 60 Years and Older, 2018–19 to 2021–22

Sensitivity analysis of VE by definition	Cases who were vaccinated, No./Total No. (%)	Controls who were vaccinated, No./Total No. (%)	Estimated VE ^a^ (95% CI), %	
			Unadjusted	Adjusted ^b^
Overall (excluding untyped)	320/1787 (17.9)	603/1787 (33.7)	57.2 (50.0 to 63.3)	63.1 (55.8 to 69.2)
PCR (only)	242/1340 (18.1)	509/1340 (38.0)	62.6 (55.2 to 68.8)	69.6 (62.5 to 75.3)
Permanent residents (only)	315/1664 (18.9)	604/1698 (35.6)	57.7 (50.5 to 63.9)	64.3 (57.2 to 70.3)
Outpatients (only)	320/1821 (17.6)	609/1821 (33.4)	57.6 (50.5 to 63.7)	63.3 (56.1 to 69.3)
Epidemic period of influenza ^c^				
Overall	316/1787 (17.7)	588/1787 (32.9)	56.6 (49.2 to 62.9)	62.1 (54.5 to 68.4)
Influenza A	309/1582 (19.5)	544/1582 (34.4)	53.7 (45.5 to 60.6)	59.8 (51.5 to 66.7)
Influenza B	7/169 (4.1)	36/169 (21.3)	84.0 (63.0 to 93.1)	85.3 (63.9 to 94.0)

Abbreviations: PCR, polymerase chain reaction; VE, vaccine effectiveness; CI, confidence interval

^a^ Calculated as (1 - odds ratio [OR]) × 100%, where OR is the odds of vaccination among cases compared with the odds of vaccination among controls

^b^ Adjusted for factors identified in Table 1 and Supplemental Table 2 as having significant bivariate associations with vaccination or case status, such as age at the time of testing, household registration, season, vaccination status in the season before inclusion, and prevalence of diabetes mellitus or hypertension

^c^ Epidemic period was defined as months each year when > 10% of all sampling was positive

## Discussion

We estimated the effectiveness of inactivated vaccination against laboratory-confirmed influenza in Chinese older adults aged ≥ 60 years to be 63.5% (95% CI, 56.3–69.5%), but estimates varied by season. These VE estimates remained robust after adjusting for potentially confounding participant demographics, clinical characteristics, and history of influenza vaccination in the previous season. Furthermore, it is reassuring and noteworthy that IIV showed better protection in people under 80 years of age, even though the VE for older people aged 80 years and above was only 45.4%

Studies have confirmed that estimates of the effectiveness of influenza vaccines vary considerably from one influenza season to another [1, 5, 6]. The effectiveness of the influenza vaccine decreases when the vaccine is mismatched or poorly matched to the prevalent strain [17]. A meta-analysis of individual participant data from a test-negative design case-control study showed that the influenza vaccine was 44.4% (95% CI, 22.6–60.0%) effective during influenza seasons in which the vaccine was matched to the prevalent strain, compared with only 20.0% (95% CI, 3.5–33.7%) effectiveness during mismatched seasons [18]. Interestingly, all four seasons examined in this study showcased more favorable VE estimates, with marginal declines observed for the 2019-20 and 2021-22 seasons. Surveillance data from the Chinese National Influenza Center showed that the vaccine strains matched poorly with pandemic influenza strains in the 2019-20 season, while better strain matches were observed in all other three seasons (see Supplemental Table 4). However, predominantly A/H3N2 strains were prevalent in the 2019-20 and 2021-22 seasons [19, 20]. Extensive research has consistently reported higher VE estimates for H1N1pdm09 and influenza B strains than A/H3N2 strains [21, 22]. Consequently, we hypothesize that estimates of VE may be influenced by the degree of match between the vaccine strain and the prevalent strain. However, we believe the prevalent strain type also has a significant effect. In addition, we think it needs to be viewed cautiously for the 2020-21 flu season VE estimate of 92.8%. This was during the severe period of COVID-19 in China when epidemic prevention measures such as wearing masks for all people blocked the COVID-19 epidemic along with the influenza epidemic. Influenza surveillance in China showed that influenza was at historically low epidemic levels that season [13, 23]. This trend was consistently observed in the surveillance data pertaining to the Ningbo region (see Supplemental Fig. 1). It has been suggested that exposures in the case and control groups may differ when the disease is at a low epidemic level, which could affect the estimation of VE values. In addition, a higher proportion of vaccinated individuals who wore masks correctly had a lower risk of infection than unvaccinated individuals [24]. There was also a greater awareness of seeking medical attention [25]. These scenarios will likely lead to overestimated VE values in case-control studies with a test-negative design

As aging ensues, the naïve B-cell and naïve T-cell reservoirs decline among older adults, resulting in a diminished capacity to mount novel immune responses to antigens [26]. This waning immunological capability profoundly impacts their immune reaction to antigenically drifted influenza viruses [17, 27]. The relatively weaker immunogenicity and protection of standard-dose inactivated vaccines in older adults than in other adults has raised concerns [1, 5–7, 27]. Consequently, the scientific community has directed considerable attention toward exploring and developing novel influenza vaccines tailored to the older adult population. A meticulous literature review included seven randomized controlled trials that collectively revealed the effectiveness of using a high-dose vaccine in reducing the risk of a laboratory-confirmed case of influenza by an impressive 24.0% (95% CI: 10.0-35.0%) in those who received the high-dose vaccine compared to those who received the standard-dose vaccine [28]. Another study evaluating the immunization efficacy of recombinant influenza vaccine in adults 50 and older showed that the recombinant vaccine was expected to be 30% more effective in protecting against laboratory-confirmed influenza compared to standard-dose inactivated vaccine [29]. In light of these compelling observations, a growing cohort of researchers has suggested the application of novel influenza vaccines, including but not limited to high-dose inactivated, recombinant, and adjuvanted vaccines, for the elderly populace to augment their immune safeguarding capabilities [5, 6, 30]. It is noteworthy that in our investigation, the immune-protective capacity of the standard antigen-content IIV showed encouraging results in mitigating influenza-related visits among the elderly aged 60 years and older. However, it is imperative to recognize that subgroup analyses revealed a significant decline in vaccine protection among those aged 80 years and over. Similar trends have been reported in previous studies. An early placebo-controlled randomized clinical trial in the Netherlands found that the effectiveness of influenza vaccination was 57% in the 60–69 age group, whereas it was only 23% in those aged 70 years and older [31]. Henrique Pott et al. also observed a lower VE of 36.8% among those aged 85 years and older, compared to 48.4% in the 65–74 age group and 52.6% in the 75–84 age group, when administered non-adjuvanted trivalent inactivated influenza vaccine [32]. Furthermore, the study compared the VE of adjuvanted and non-adjuvanted vaccines, revealing that adjuvanted vaccines generally offered superior protection, with the most significant increase observed in the 85 years and older group. Notably, after adjusting for factors such as clinical frailty scores, the VE of both non-adjuvanted and adjuvanted vaccines remained relatively stable among those under 85 years of age. However, among those aged 85 years and older, the VE of non-adjuvanted vaccines remained largely unchanged (36.8% vs. 33.9%), while the VE of adjuvanted vaccines showed a notable increase (49.8% vs. 57.0%) [32]. Given these findings, it seems imperative to develop and utilize novel vaccines specifically tailored for elderly individuals of advanced age, thereby alleviating the burden of influenza in this vulnerable population

Notably, our study possesses inherent limitations associated with test-negative study designs, wherein controlling for case and control exposure remains challenging [33]. It is imperative to acknowledge that despite our meticulous efforts to select cases and controls from the same source population, along with the independent selection of controls irrespective of influenza vaccination status, the presence of residual confounding factors cannot be entirely ruled out. Nucleic acid testing is the gold standard diagnostic approach for detecting influenza due to its remarkable reliability [2], whereas antigen testing exhibits lower sensitivity [7, 34]. Adhering to a real-world data framework, our investigation embraced antigen testing and nucleic acid testing as diagnostic criteria, potentially introducing spurious estimations of VE. However, upon restricting the case group to nucleic acid-positive patients or the control group to nucleic acid-negative patients, our calculations indicated no significant alterations in the VE estimates. Regrettably, data limitations hindered our access to testing results for pathogens beyond influenza in this study, imposing restrictions on comprehensive assessments. Motoi Suzuki et al. have posited that the simultaneous circulation of non-influenza respiratory viruses may introduce uncertainties in estimating the protective effect of influenza vaccines in test-negative case-control studies [35]. However, the available evidence surrounding this issue remains elusive and inconclusive. A prospective case-control study by Heath Kelly et al. found that estimates of vaccine protective effect were higher when the control group was limited to patients who tested positive for respiratory viruses other than influenza. In contrast, the opposite was confirmed when the control group was defined as patients who tested negative for flu [36]. However, certain studies have posited that the control group may consist of patients testing negative for influenza for test-negative case-control investigations, with other pathogen detections negligibly impacting the final VE estimate [37, 38]. Thus, the optimal definition and composition of the control group in the context of test-negative designs remains subject to ongoing scientific discourse and warrants further exploration. Some test-negative design studies gauging vaccine efficacy restrict the study period to peak influenza activity [39]. In congruence with the survey conducted by Ho et al. [40], our analysis integrated year-round surveillance data to evaluate influenza VE. However, Jackson et al. suggested that cases occurring outside the epidemic period should be excluded from such investigations due to potential incongruity with subjects exposed during the typical epidemic season [41]. To address this concern, we judiciously adjusted for potential confounders arising from variant inclusion periods during the vaccine effect analysis. Concurrently, we conducted a sensitivity analysis by confining the study period exclusively to the peak of the influenza epidemic. Impressively, the vaccine protection effect estimates obtained from this supplementary analysis remained consistent with the primary analysis outcomes. Regrettably, due to the unavailability of live attenuated vaccines for adults in China and the paucity of elderly individuals receiving quadrivalent inactivated vaccines within our study population, we could not derive VE values for these specific vaccine types within this demographic. Furthermore, despite our study encompassing a considerable number of cases and controls, certain subgroups may have exhibited small sample sizes, potentially leading to the detection of spurious events during sensitivity and subgroup analyses, thereby possibly compromising the statistical power to detect such events. Finally, we acknowledge the potential impact of immunocompromised status and frailty on vaccine response and influenza susceptibility among the elderly. However, our current clinical records lack these assessments, limiting our ability to comprehensively evaluate their influence on vaccine effectiveness. To mitigate this limitation, we have included chronic underlying disease status as a proxy measure of health among the elderly. Nevertheless, we recognize the impact of this limitation and aim to improve data collection in future studies. While our study carries notable limitations, accounting for various confounding factors and striving for robustness in our analyses, these constraints underscore the need for further research to comprehensively elucidate the nuances of VE in combating influenza within diverse populations.

## Conclusion

Inactivated influenza vaccination significantly reduces influenza-associated visits in the elderly despite the limited protective effect in advanced age. Our data support the recommendation of annual influenza vaccination for all older adults in the absence of known contraindications.

### Electronic supplementary material

Below is the link to the electronic supplementary material.

Supplementary Material 1

## Data Availability

Please contact the corresponding author for data requests.

## Abbreviations

VE: vaccine effectiveness
OR: odds ratio
CI: confidence interval
IIV: inactivated influenza vaccine
PCR: polymerase chain reaction
ILI: influenza-like illness
NRHIP: Ningbo Regional Health Information Platform
IQR: interquartile ranges
COVID-19: coronavirus disease 2019
