# Supplementary material for: Effectiveness of inactivated influenza vaccine against laboratory-confirmed influenza among Chinese elderly: a test-negative design
Source: BMC Geriatr. 2024 May 7;24:404. doi: 10.1186/s12877-024-05003-3 (PMC11077745; doi:10.1186/s12877-024-05003-3)
Supplement: Supplementary file 1 — Supplementary Material 1 [file 12877_2024_5003_MOESM1_ESM.doc]

**Supplemental Table 1. International Classification of Diseases (ICD) Codes Definition of “Influenza-like illness”**

| Disease | ICD code |
| --- | --- |
| Cold | J00 |
| Sinusitis | J01, J32 |
| Pharyngitis | J02 |
| Laryngitis, tracheitis or laryngotracheitis | J04 |
| Upper respiratory tract infections | J06.8 or J06.9 |
| Influenza | J09-J11 |
| Pneumonia | J12-J18 |
| Acute bronchitis, bronchiolitis (not specifically acute or chronic), obstructive bronchitis | J20, J40, J44.8 |
| Fine bronchitis, capillary bronchitis | J21 |
| Acute lower respiratory tract infections, nonspecific | J22 |
| Chronic obstructive pulmonary disease combined with acute lower respiratory tract infection | J44.0 |
| Chronic obstructive pulmonary disease with acute exacerbation | J44.1 |
| Cough | R05 |
| Pleurisy | R09.1 |

**Supplemental Table 2. Demographic and Clinical Characteristics of Vaccinated** and Unvaccinated Subjects

| Characteristic |  | Total (N=3650) | Vaccinated (N=931) | Non-vaccinated (N=2719) | P value ^a^ |
| --- | --- | --- | --- | --- | --- |
| Age at presentation, y | M (IQR) | 67 (9) | 72 (8) | 65 (8) | <0.001 |
| Age group, y |  |  |  |  | <0.001 |
|  | 60 to <70 | 2392 (65.5) | 370 (39.7%) | 2022 (74.4%) |  |
|  | 70 to <80 | 934 (25.6) | 458 (49.2%) | 476 (17.5%) |  |
|  | 80+ | 324 (8.9) | 103 (11.1%) | 221 (8.1%) |  |
| Sex |  |  |  |  | 0.236 |
|  | Female | 1892 (51.8) | 467 (50.2%) | 1425 (52.4%) |  |
|  | Male | 1758 (48.2) | 464 (49.8%) | 1294 (47.6%) |  |
| Household registration | Yes | 3362 (92.1) | 919 (98.7%) | 2443 (89.8%) | <0.001 |
| Influenza season |  |  |  |  | <0.001 |
|  | 2018-2019 | 436 (12.0) | 17 (1.8%) | 419 (15.4%) |  |
|  | 2019-2020 | 578 (15.8) | 42 (4.5%) | 536 (19.7%) |  |
|  | 2020-2021 | 180 (4.9) | 34 (3.7%) | 146 (5.4%) |  |
|  | 2021-2022 | 2456 (67.3) | 838 (90.0%) | 1618 (59.5%) |  |
| Vaccination status in the previous season | Yes | 538 (14.7%) | 345 (37.1%) | 193 (7.1%) | <0.001 |
| Medical history |  |  |  |  |  |
| Diabetes mellitus | Yes | 432 (11.8) | 145 (15.6%) | 287 (10.6%) | <0.001 |
| Cerebrovascular diseases | Yes | 71 (2.0) | 20 (2.1%) | 51 (1.9%) | 0.603 |
| Hypertension | Yes | 1230 (33.7) | 426 (45.8%) | 804 (29.6%) | <0.001 |
| Tumors | Yes | 92 (2.5) | 23 (2.5%) | 69 (2.5%) | 0.910 |

^a^ Cases and controls were compared using the Pearson χ2 test or Fisher’s exact test for categorical variables and the Wilcoxon rank sum test or t-test for continuous variables.

Abbreviations: M (IQR), median (interquartile range).

**Supplemental Table 3. Subgroup Analyses of Vaccine Effectiveness of Inactivated Influenza Vaccine among People Aged 60 Years and Older, 2018-19 to 2021-**22

| Effectiveness by Characteristic | Cases who were vaccinated, No./Total No. (%) | Controls who were vaccinated, No./Total No. (%) | Estimated VE (95% CI), % | |
| --- | --- | --- | --- | --- |
|  |  |  | Unadjusted | Adjusted |
| Before the COVID-19 epidemic | 2/218 (0.9) | 15/218 (6.9) | 88.1 (46.7 to 97.3) | 84.2 (26.1 to 96.6) |
| Influenza A | 2/176 (1.1) | 6/176 (3.4) | 67.4 (-63.6 to 93.5) | 52.5 (-170.9 to 91.7) |
| Influenza B | 0/4 (0) | 1/4 (25.0) | - | - |
| During the COVID-19 epidemic | 318/1607 (19.8) | 596/1607 (37.1) | 58.5 (51.3 to 64.7) | 61.9 (54.4 to 68.2) |
| Influenza A | 309/1411 (21.9) | 541/1411 (38.3) | 54.9 (46.8 to 61.8) | 58.9 (50.4 to 65.9) |
| Influenza B | 9/196 (4.6) | 55/196 (28.1) | 87.7 (74.2 to 94.1) | 87.2 (72.4 to 94.1) |

Abbreviations: VE, vaccine effectiveness; CI, confidence interval.

**Supplemental Table 4. Match between Influenza Epidemic Strains and Vaccine Strains by Season**

| Season | Subtype | Vaccine strain | Epidemic strain | Proportion | Matching |
| --- | --- | --- | --- | --- | --- |
| 2018/19 | A/H1N1 | A/Michigan/45/2015 (H1N1)pdm09-like virus | A/Michigan/45/2015-like virus | 97.6% | Yes |
|  | A/H3N2 | A/Singapore/INFIMH-16-0019/2016 (H3N2)-like virus | A/Singapore/INFIMH-16-0019/2016(cell) | 97.0% |  |
|  |  |  | A/Singapore/INFIMH-16-0019/2016(chicken embryo) -like virus | 78.5% |  |
|  | B/Victoria | B/Colorado/06/2017-like virus (B/Victoria lineage) | B/Colorado/06/2017-like virus | 42.4% |  |
|  | B/Yamagata | B/Phuket/3073/2013-like virus (B/Yamagata lineage) | - | - |  |
| 2019/20 | A/H1N1 | A/Brisbane/02/2018 (H1N1)pdm09-like virus | - | - | No |
|  | A/H3N2 | A/Kansas/14/2017 (H3N2)-like virus | A/Kansas/14/2017(cell)-like virus | 8.3% |  |
|  |  |  | A/Kansas/14/2017(chicken embryo)-like virus | 3.8% |  |
|  | B/Victoria | B/Colorado/06/2017-like virus (B/Victoria lineage) | B/Colorado/06/2017-like virus | 16.9% |  |
|  | B/Yamagata | B/Phuket/3073/2013-like virus (B/Yamagata lineage) | - | - |  |
| 2020/21 | A/H1N1 | A/Guangdong-Maonan/SWL1536/2019(H1N1) pdm09-like virus | - | - | Yes |
|  | A/H3N2 | A/HongKong/2671/2019 (H3N2)-like virus | - | - |  |
|  | B/Victoria | B/Washington/02/2019- like virus (B/Victoria lineage) | B/Washington/02/2019-like virus | 36.3% |  |
|  | B/Yamagata | B/Phuket/3073/2013-like virus (B/Yamagata lineage) | - | - |  |
| 2021/22 | A/H1N1 | A/Victoria/2570/2019 (H1N1)pdm09-like virus | - | - | Yes |
|  | A/H3N2 | A/Cambodia/e0826360/2020 (H3N2)-like virus | A/Cambodia/e0826360/2020(cell) | 89.0% |  |
|  |  |  | A/Cambodia/e0826360/2020(chicken embryo) | 86.5% |  |
|  | B/Victoria | B/Washington/02/2019 (B/Victoria lineage)-like virus | B/Washington/02/2019--like virus | 33.8% |  |
|  | B/Yamagata | B/Phuket/3073/2013 (B/Yamagata lineage)-like virus | - | - |  |

Matching was defined as the proportion of prevalent strains that were similar to the corresponding annual vaccine strain being not less than 30%.

The source of data is the results of antigenicity analysis published annually in the Influenza Weekly Report of Chinese National Influenza Center (Available at: <https://ivdc.chinacdc.cn/cnic/zyzx/lgzb/>).


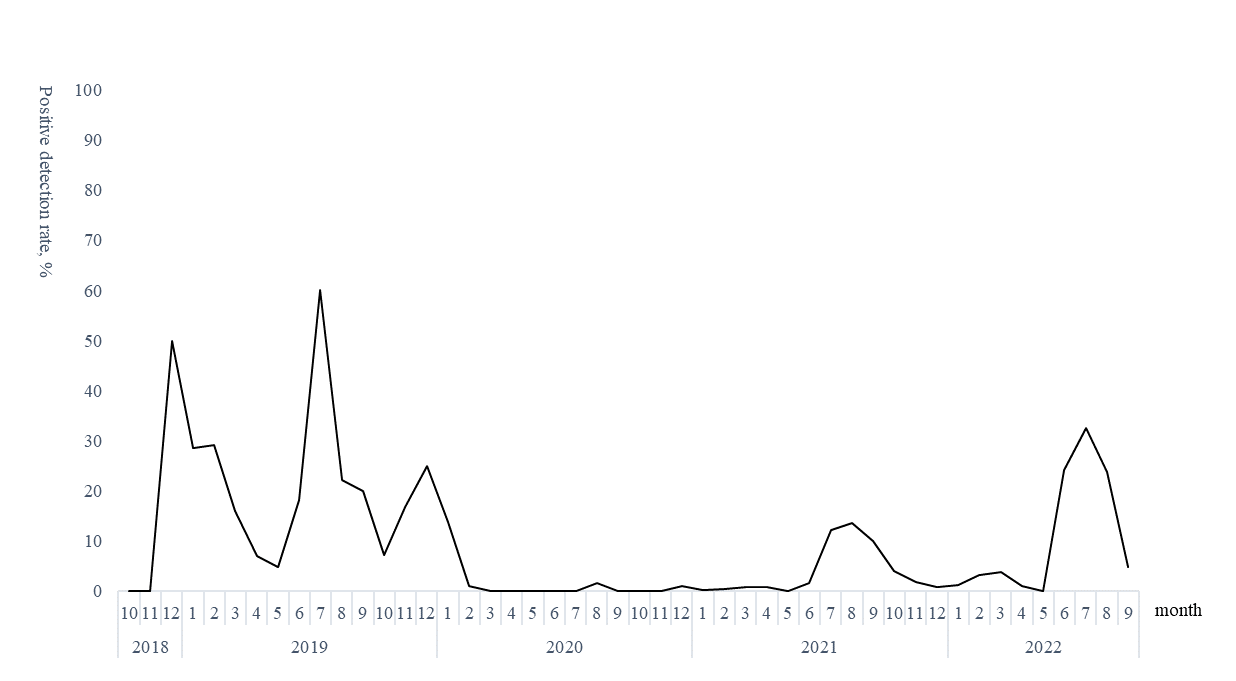


**Supplemental Figure 1.** Positive Detection of Influenza in Ningbo, 2018-19 to 2021-22
